# Psychological resilience of emergency nurses during COVID-19 epidemic in Shanghai: A qualitative study

**DOI:** 10.3389/fpubh.2022.1001615

**Published:** 2022-09-16

**Authors:** Jinxia Jiang, Yue Liu, Peng Han, Pengjia Zhang, Haiyan Shao, Hu Peng, Xia Duan

**Affiliations:** ^1^Emergency Department, Shanghai Tenth People's Hospital, School of Medicine, Tongji University, Shanghai, China; ^2^Nursing Department, Shanghai First Maternity and Infant Hospital, School of Medicine, Tongji University, Shanghai, China

**Keywords:** COVID-19, psychological resilience, coping strategies, emergency nurses, epidemic, qualitative research

## Abstract

**Background:**

In early 2022, an outbreak of Coronavirus Disease 2019 (COVID-19) occurred in Shanghai, China. The spread of the epidemic put a large amount of stress on the local healthcare system, especially emergency nurses (ENs), which may affect their well-being and performance. Enhancing the psychological resilience of ENs during COVID-19 pandemic may improve job satisfaction, retention, and public health emergency response. However, few studies have researched the perception and psychological resilience of ENs during COVID-19 pandemic.

**Objective:**

To understand ENs' perception and psychological resilience and their coping strategies with adversity during COVID-19 in Shanghai, as well as factors associated with psychological resilience.

**Methods:**

This qualitative study was conducted using a phenomenological approach. A total of 17 ENs from a 3rd level hospital in Shanghai were selected using a method of purposive sampling. Between April and May 2022, in-depth semi-structured interviews and Colaizzi seven-step method were performed for data collection and analysis.

**Results:**

The investigation discovered three themes and nine subthemes. The first theme is “risk factors.” Risk factors for ENs to remain resilience are sudden multiplication of workload, stressful of screening of infected patients, and the support nurses being unfamiliar with the procedure. The second theme is “promoting factors.” ENs emphasized the importance of management assurance and humanistic care, as well as social support. They recognized adversity and resilience, and used self-management strategies to cope with the situation. The third theme is “motivated by altruism.” ENs were driven by altruism to respond to adversity with a positive attitude. They realized their self-worth by helping patients with a sense of sacred mission.

**Conclusions:**

Psychological resilience is not a stable psychological characteristic but a constantly changing process that is affected by internal and external factors. Enhancing resilience of ENs during the COVID-19 pandemic may improve work satisfaction, retention, and public health emergency response. Adequate preparation before a pandemic, reasonable arrangement, a trustworthy working atmosphere, encouragement and improvement of individual and collective strategies for nurses to cope with adversity, timely rewards, and nurse empowerment, as well as counseling and training, can be used to enhance psychological resilience of ENs.

## Introduction

Coronavirus Disease 2019 (COVID-19) is a highly contagious infectious disease that has become a global public health emergency (1, 2). Most patients with COVID-19 are asymptomatic or have mild symptoms, such as cough and fever; pneumonia, sepsis, and acute respiratory distress syndrome may occur in severe cases. So far (June 2022), more than 555,446,890 infections and 6,353,692 deaths have been registered worldwide (3). Before March 2022, there were only 393 confirmed cases of COVID-19 in Shanghai, China. However, from March to June 2022, the number of infections in Shanghai reached 650,047, with a cumulative death toll of 595 (4).

The city of Shanghai, with its 16 regions, is the most populous urban area in China and one of the 10 most populous cities in the world (5). At the end of 2021, there were 24,894,300 residents living in Shanghai. Also, the city has a migrant population of more than 10 million (6). High population density and mobility present a big challenge for epidemic prevention and control. In order to optimize patient care with the best resource allocation, COVID-19 patients in Shanghai have been treated centrally at the Shanghai Public Health Clinical Center from early 2020. Centralized treatment helps existing medical resources operate normally, reduce the spread of outbreaks, and minimize the risk of nosocomial infection. It also improves diagnosis and treatment rates, thus reducing the rate of serious illness and mortality from COVID-19 (7). In addition, Shanghai Public Health Clinical Center's headquarters in Shanghai's Jinshan District is surrounded by a nearly 1/3 square kilometer wooded area to ensure the safety of nearby residents (8). Furthermore, in 2003, the Chinese government built the Xiaotangshan Hospital for SARS patients (9). However, a total of 58,137 confirmed cases of COVID-19 and 591,910 asymptomatic infections were reported in Shanghai between March 1, 2022, and June 30, 2022 (10), which has put some pressure on the healthcare system. With the surge of infections, emergency departments (EDs) have been under tremendous pressure (11).

Emergency Medical Service System (EMSS) in Shanghai operates independently from other major medical institutions, unlike in some other Chinese cities. Shanghai Medical Emergency Center (Shanghai “120”) is responsible for the emergency services of 16 regions. It transports patients to the nearest hospital or the hospital of the patient's choice. Although Shanghai “120” organized a special system connecting all hospital EDs through telephone, processing a high load of complex information may be difficult. In addition, transferring infected cases screened by hospitals to square-cabin hospitals and designated hospitals is challenging. Among them, square-cabin hospitals were used to isolate asymptomatic infected individuals, and designated hospitals were used to admit confirmed cases of COVID-19 who needed treatment. The overwhelming number of patients with COVID-19 in need of transfer led to a shortage of transport capacity for Shanghai “120,” and many infected cases were sent to EDs. As medical resources were squeezed, EDs were under increasing pressure for treatment. As the number of infections continues to rise, the Shanghai government has recently (April 30, 2022) built 40 designated hospitals with 23,000 beds available and more than 100 square-cabin hospitals with 160,000 beds (10, 12). This, in turn, led to a shortage of nurses working in DEs.

Emergency nurses (ENs) are always at the forefront of the battle against COVID-19, having a crucial role in identifying suspected and confirmed patients (13). In addition, they are responsible for implementing efficient infection control measures and preventing the epidemic from spreading further (14). After the epidemic outbreak, some nurses remained in ED, while others went to the square-cabin hospitals and the designated hospitals. In order to relieve the pressure on ED, the nursing department mobilized the human nursing resources.

The effects of stressful situations on ED staff can be profound (11). Depression, anxiety, work burnout, post-traumatic stress disorder (PTSD), and other negative emotions have been reported among nurses during the COVID-19 epidemic (15). During the Wuhan pandemic in early 2020, more than 70% of nurses were experiencing psychological issues (16). Also, Bohlken et al. (17) discovered that 2.2 to 14.5% of medical staff had psychological problems under the COVID-19 pandemic. Shen et al. noticed psychological pressure, including sleeplessness, sobbing, and attempted suicide among nurses working in the critical care medicine department of Wuhan Pulmonary Hospital, a designated hospital for the treatment of severe patients with COVID-19 (18). Other studies pointed out that the psychological pressure among nurses is strongly associated with excessive working hours, inadequate personal protective equipment (PPE), fear of infection for themselves and their families, and social stigmatization (19, 20). A challenging, exceedingly chaotic, and risky work environment in ED during the COVID-19 epidemic increased the physical burden, psychological distress, and moral anguish of ENs. Also, psychological resilience has been considered an advantageous ability for coping with psychological issues (21), which is necessary for the endurance of ENs during this adversity.

Psychological resilience is described as the psychological characteristic that facilities adapt to adversity, catastrophes, traumatic events, or severe cause of stress, which is well-valued as it alleviates stress in the workplace (22). In addition, resilience can improve the quality of care and patient satisfaction (22). In contrast to the traditional view of resilience as an inherent psychological characteristic of the individual, Richardson (23) described resilience as a dynamic and balanced process that is disrupted and reintegrated by the action of various factors. Evidence suggests that characteristics such as adaptability, control, coping, hope, self-efficacy, and skill identification are crucial for resilience in nurses (24). In addition, a US study discovered a positive correlation between resilience and social support (25).

Psychological resilience is critical for ENs, given the danger and pressure they face during their everyday activities, and especially during outbreak control and patient interaction. Many studies have highlighted the relationship between psychological resilience and nurse satisfaction, burnout, pressure, and turnover (26–28). However, qualitative research on the psychological resilience of ENs during the COVID-19 epidemic is scarce. Thus, the purpose of this study was to understand ENs' perception and psychological resilience and their coping strategies with adversity during COVID-19 in Shanghai, as well as factors associated with psychological resilience.

## Methods

### Study design

A qualitative study using the phenomenological approach was selected to explore the psychological resilience of ENs during the COVID-19 epidemic in Shanghai. This approach is an inductive and descriptive method that facilitates understanding the human complexity experience (29) and has been widely used in the field of nursing in recent years (30).

### Ethical considerations

The Institutional Review Committee of Shanghai Tenth People's Hospital granted ethical approval (No: 22KN08). All participants were informed of the purpose of the study and that all conversations would be recorded. Participants were volunteers and could withdraw from the study at any time. The researcher explained the purpose and voluntary of the study in detail to the participants via email. Each participant read the informed consent carefully and signed an Electronic Consent Statement prior to the start of the interview. The privacy of all participants was protected by using numbers instead of participant names (e.g., “participant 1 “), while other identifying information was removed.

### Participants

A total of 17 ENs from a 3rd level hospital in Shanghai were included in the study. The inclusion criteria were: (a) at least 3 years of work experience in ED; (b) at least 1 month of work experience in ED during the COVID-19 pandemic; (c) participants were able to adequately express emotional experience; (d) willingness to participate voluntarily. The exclusion criteria were: (a) experiencing serious emotional disturbances; (b) ENs infected with COVID-19 who need a break. The type of sampling method chosen for this research was purposive sampling (31). In qualitative research, the sample size depends on the purpose of the study, the quality of the information, and the type of sampling strategy used. We determined the required sample size by conducting interviews with ENs who met the inclusion criteria until data saturation was achieved and no new themes were generated (32). The participants' detailed characteristics are listed in Table 1.

**Table 1:** Participant characteristics (*n* = 17).

**Characteristics**	***N* (%) or Mean (SD)**
**Sex**	
Male	6 (35.3)
Female	11 (64.7)
Age, mean (range)	30.41 (23~46)
Years of employment	8.35 (3.76)
**Education level**	
Diploma	4 (23.5)
Baccalaureate Degree	12 (70.6)
Master Degree	1(5.9)
**Marital status**	
Married	9 (52.9)
Single	8 (47.1)
**Place of birth**	
Jiangsu Province	5(29.4)
Anhui Province	2(11.8)
Henan Province	1(5.9)
Hebei Province	1(5.9)
Sichuan Province	2(11.8)
Shanghai City	6(35.2)

### Data collection

Because the participants worked in ED and were isolated in different hotels, traditional face-to-face interviews were not conducted. To ensure the credibility and validity of the interviews, WeChat video call was applied. Between April and May of 2022, semi-structured interviews were conducted online during the participants' brake. The interview questions, which are presented in Table 2, were derived from prior studies (20, 33, 34). To ensure the validity of the questions, a pilot interview was conducted with two ENs before the formal interview. The pilot interview was just a test, so it wasn't used in the analysis. Researchers received qualitative research system training, and all were with rich experience in qualitative research. The interviews were audio recorded and lasted some 30 min to 1 h. Two co-authors conducted the interviews in Chinese; one conducted the online interviews, and the other recorded and analyzed the participants' responses in detail, including the non-verbal behaviors and expressions during each interview. Every participant received a small gift (course credit or water bottle) as a reward for participation. All participants completed the interviews. The information data saturation was achieved and verified after 17 interviews.

**Table 2:** The interview questions.

**No**.	**Question**
1	What challenges are you currently facing at work?
2	What is the greatest difficulty or obstacle you have encountered so far?
3	What kind of impact did your current position have on you?
4	How did you deal with these obstacles or challenges?
5	What additional perception and experience did you have?
6	During the epidemic, have you considered quitting the nursing position? If the answer is “Yes,” why?

### Data analysis

The audio recordings were transcribed word-for-word and analyzed within 24 h following the interview. Themes and subthemes were independently extracted from the data by two experienced researchers using Colaizzi's phenomenological seven-step method (29) (Table 3).

**Table 3:** Colaizzi's phenomenological seven-step method framework.

**Step**	**Data analysis procedure**
1	All interviews were recorded using audio equipment and transcribed within 24 h.
2	Significant sentences that directly related to the viewpoints and experiences of ENs were re-read, underlined, and extracted manually during the fight against the COVID-19 epidemic.
3	Meanings from all significant statements were summarized. In this process, the composition of meaning was reviewed by two PhDs with extensive experience in qualitative research, and graduate students with experience in qualitative research also participated in qualitative research.
4	The summarized meanings were classified into theme clusters. The researchers compared the theme clusters with the original data several times to determine consistency.
5	Nine subthemes were identified to describe ENs' perception and psychological resilience during COVID-19 epidemic in Shanghai and the researched phenomenon was characterized in detail.
6	Similar sub-themes were organized into larger clusters, and three main themes were obtained. The fundamental framework of the viewpoints and experiences of ENs during COVID-19 epidemic was described.
7	The fundamental framework was returned to the participants to verify whether the content was consistent with their perception and experiences during the COVID-19 epidemic.

In the first phase, all interviews were recorded using audio equipment and transcribed within 24 h.

In the second phase, important statements directly related to the viewpoints and experiences of ENs during the COVID-19 epidemic were re-read, underlined, and extracted and numbered manually.

In the third phase, meanings from all significant statements were summarized. In this process, the composition of meaning was reviewed by two PhDs with extensive experience in qualitative research, and graduate students with experience in qualitative research also participated in qualitative research.

In the fourth stage, the summarized meanings were classified into theme clusters. In this process, the researchers compared the theme clusters to the original data to determine agreement, and repeated these processes several times.

In the fifth stage, nine subthemes were identified to describe ENs' perception and psychological resilience during COVID-19 epidemic in Shanghai and exhaustive descriptions were developed.

In phase six, similar sub-themes were organized into larger clusters, and three main themes were obtained.

In phase seven, essential structures were returned to the participants to verify whether the content was consistent with their perception and experiences during the COVID-19 epidemic.

In case of disagreement, a consensus was reached by discussion. All participants consented to be contacted once more and supplied their phone numbers to the researchers.

### Methods of rigor

Four criteria defined by Lincoln and Guba were used to ensure methodological rigor: credibility, confirmability, dependability, and transferability (35). To improve the credibility of the data, the final results were sent to all participants for confirmation and approval. Confirmability was ensured through a clear description of the study context, sampling, and the process for data collection and analysis. In terms of dependability, two external experts experienced in qualitative research reviewed the decision cues as well as the study's findings and conclusions. In terms of transferability, the sample we recruited varied in length of employment, level of competence, gender, and age. This reporting was guided by the Comprehensive Standard for Reporting Qualitative Research (COREQ) (36).

## Results

**Figure 1:**
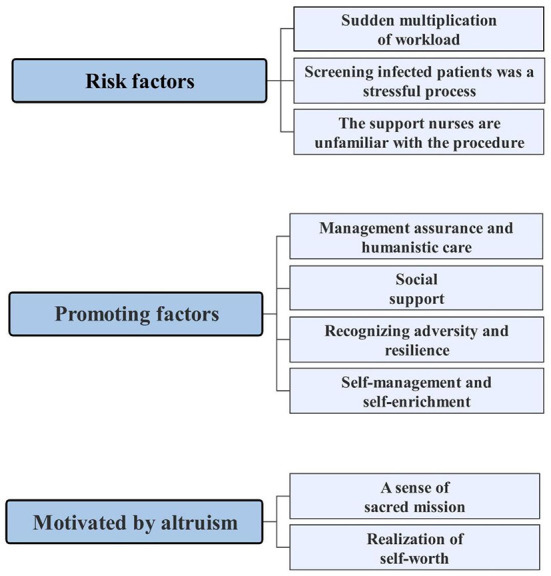
Themes and subthemes.

Semi-structured interviews provided a platform for capturing the perception and psychological resilience of ENs and the ways they coped with adversity during the COVID-19 pandemic in Shanghai. Three themes emerged from the data: (a) risk factors, (b) promoting factors, and (c) motivated by altruism. Figure 1 illustrates the three themes and subthemes summarized in this research.

### Risk factors

#### Sudden multiplication of workload

COVID-19 outbreak, a surge of emergency patients, strict isolation measures, and a chaotic work environment resulted in a heavy workload and increased working hours for ENs. “*In Shanghai, ambulances transported at least 95 patients to ED daily. We analyzed patients' antigens and nucleic acids on a daily basis, taking samples and cleaning the environment and surfaces. All of these tasks were new and exhausting..*.” (Participant 9). “*We had to do all the charging, registration, and medication collection, and there unexpected problems just kept coming up, which was very stressful*.” (Participant 13). “*We would have 8 hours of continuous work. I felt too tired to talk. We were often too busy and had no more than a half-hour lunch break. After all, putting on and removing PPE is laborious. The 6-hour shift slightly improved things (relieved)*.” (Participant 16). “*There were no nurse assistants. We did everything, from cleaning patients' bodies to changing oxygen cylinders, feeding, and changing nappies… saying that we were very busy is an understatement*.” (Participant 2).

In addition, most participants had increased difficulty working while wearing PPE, including nose discomfort, headaches, tiredness, and limited movement. As the pandemic continued, lack of PPE prevented ENs from changing clothes, eating, drinking, and using the washroom during shifts. “*My face shield was cloudy; it blocked my vision, and prevented me from providing the care that my older adult patient deserved*. *Preforming venipuncture with two layers of gloves was challenging*.” (Participant 6). “*Sometimes emergency specialist doctors were sent out to support, and doctors came from the wards. Families argued with us because we took longer time to put on the PPE*.” (Participant 5).

#### Screening infected patients was a stressful process

The “closure” of Shanghai and the high contagiousness of COVID-19 induced fear and uncertainty among individuals, including healthcare professionals. ENs are under pressure to identify infected patients and are worried they might get infected. “*Three nurses at the emergency entrance were responsible for tested antigens, reviewing nucleic acid reports, communicating big data travel card, and health QR codes every day while remaining concerned about triage safety*.” (Participant 8). “*Having everyday contact with positive patients is dangerous. Several of my coworkers were infected, and I'm worried I'll be next (pouted)*.” (Participant 1).

Meanwhile, our interviews revealed that inadequate training, poor procedures, and ineffective communication among hospital management made it difficult for ENs to screen infected patients. “*Some patients with fever whose vital signs were unstable may be were turned away from the fever clinic and sent to ED, which is more troublesome*.” (Participant 11). “*When patients tested positive for antigens, ambulances brought them to our ED rather than to a designated hospital. Future transfers of such patients required take time and effort*.” (Participant 3).

It is worth noting that screening positive patients not only puts psychological pressure on ENs, but also affects their physical health and comfort. “*Every day, the workgroup needed to notify the infected patients. I eventually started to feel numb... the frequency of disinfection increased from 3 times a day to 6 times a day. Even when I was exhausted, I could not sleep. Sometimes, I'd wake up at midnight (shook head).”* (Participant 7).

#### The support nurses are unfamiliar with the procedure

Although hospital management has improved the deployment of human nursing resources and the emergency staff ladder, there are still shortcomings. The hospital's management dispatched transdisciplinary nurses (TNs) to ED support to relieve the pressure of high traffic. However, many TNs have never worked in the respiratory department, infection department, emergency department, or intensive care units. Due to a lack of role clarity and unfamiliarity with the work content and environment, the pressure on ENs remains unaddressed (37). “*There was once a large rescue, and the new nurses sent to help could not do anything. I managed the airway and set up IVs and urethral catheterization, and monitored patients. In addition, I was responsible for coordinating the rescue efforts. It's cracking me up...(frowned)*” (Participant 5). “*The new nurse was not able to draw arterial blood gas, so I was busy enough (sighed).”* (Participant 17). “*We had few nurses in the ED, and the nurses who came to support us were not familiar with the process, since the emergency nurse station operating system and the laboratory system were different from their ward. I kept teaching them while I was busy with other things, which was very chaotic*.” (Participant 14).

Participants indicated that working with an ever-changing team in ED made teamwork difficult. “*Weekly, support nurses arrived, became familiar with the process, and left. It came to be exhausting having to work with new partners every week*.” (Participant 3).

### Promoting factors

#### Management assurance and humanistic care

Individuals mobilize many promoting factors to maintain resilience against these risk factors, including management assurance and humanistic care. “*Hospital management arranged a hotel for high-risk staff during the epidemic. We were also given a special work allowance for epidemic prevention and control. We felt like they cared*.” (Participant 4). “*The supply department delivered milk, chocolate, and more foods. Caring organizations distributed donated drinks, health food, vitamins, instant noodles, and female sanitary products*.” (Participant 15).

In the face of adversity, the head nurse has the power to set the example, which is essential to maintain resilience. “*The head nurse raced alongside us on the front lines, and we felt we had someone to fall back on*.” (Participant 10).

#### Social support

The emotional experience of an individual feeling respected, supported, and understood in society usually comes from their primary sources of social support, which for ENs were family and peers. “*To avoid infecting my family, I haven't been home in 30 days. But every time I hear from them and see them on a WeChat video call, they give me great encouragement. My son repeatedly says: You're so great, Mom! (smiled)*.” (Participant 2).

In particular, the peers in the front-line team support and encourage each other, which is an important factor that increases resilience among ENs. Additionally, ENs emphasized the significance of the developed cohesion with their peers and team resilience was developed. “*Colleagues created a short video showing our day's work. Wow, as soon as it was sent to WeChat moments, I watched it with tears in my eyes and felt great afterwards (gave a thumbs up)*.” (Participant 14). “*We often encouraged and complained to one another, which made the atmosphere much more relaxed*.” (Participant 8). “*We are like chicks protected by the head nurse, receiving all kinds of care*.” (Participant 11). “*My colleague is a traditional Chinese medicine specialist nurse. She helped me with ear acupoint pressing beans and auricular point acupressure (APA), and I felt better*.” (Participant 9).

#### Recognizing adversity and resilience

Our data suggested that ENs have a clear perception of resilience and adversity. Others viewed “psychological resilience” as a dynamic process of overcoming adversity. At the same time, some nurses viewed it as a positive trait. For these nurses, resilience meant perseverance in facing the epidemic's challenges. “*In health assessment, we discussed problematic and emotional coping styles for stress. I think I'm in the former category, as I could handle problems calmly and objectively (laughed). I'm more optimistic because I believe all problems can be resolved, and we are not alone*.” (Participant 7). “*Those are tough times, but it is the same for all. I believe we will overcome the epidemic eventually*. “ (Participant 1).

#### Self-management and self-enrichment

Maintaining resilience requires engaging in self-management and self-enrichment, as well as maintaining an active and healthy lifestyle in the face of adversity, which helps effectively fight the epidemic. “*After work, a few friends grouped online to play games, which would really relax me*.” (Participant 6). “*I watched the TV serials that I usually don't have time to watch, which was fun (smiled). We now have a 6-hour shift, which is enough to view the Wechat moments, read some news, watch Tik Tok, and listen to music*.” (Participant 12). “*Food pleases my taste buds and soothes my mood, so I group purchased it on my phone (laughed)*.” (Participant 1). “*I don't have time to think about it. I'll write my undergraduate thesis and then prepare for the qualification exam*.” (Participant 2). “*Playing my favorite music, especially piano. My mind felt renewed after immersing in music*.” (Participant 7). “*After work, I would read books using Kindle and enjoy some ancient poems and euphemisms of classical literature*.” (Participant 9).

### Motivated by altruism

#### A sense of sacred mission

The mission of the profession motivates nurses to respond to stress and challenges from adversity in a positive manner, with all respondents expressing a strong sense of mission. This was in contrast to the anxiety and emotional turmoil of the early stages. “*As an “angel in white”, I must fight the virus, despite its difficulty. I take pride in the fact that I never backed down*.” (Participant 5). “*In fact, I was considering resigning before. When the epidemic happened, I withdrew my resignation and joined epidemic prevention and control*.” (Participant 9). “*I'm an eight-year Chinese Communist Party member. I should step up in a crisis..*.” (Participant 12).

#### Realization of self-worth

Altruism is motivated by a desire to enhance the welfare of others. Most interviewees indicated that they realized their self-worth by helping patients, and participating in epidemic prevention and control. “*Helping others is fulfilling the original purpose of my profession (smiled)*.”(Participant 1). “*It's really gratifying that we were able to break through the virus and save lives. It was really hard but it made me happy*” (Participant 7). “*We must help our patients as they depend on us*.” (Participant 10).

## Discussion

The purpose of this study was to understand ENs' perception and psychological resilience and their coping strategies with adversity during COVID-19 in Shanghai, as well as factors associated with psychological resilience. Three themes emerged: (a) risk factors, (b) promoting factors, and (c) motivated by altruism. Consistent with previous studies (20, 38, 39), it was determined that ENs faced many challenges, particularly in the early stages of a pandemic, such as sudden multiplication of workload, stressful screening of infected patients, and the support nurses being unfamiliar with the procedure, which are all considered risk factors affecting resilience. In addition, it was discovered that ENs collectively or individually experienced an increasing number of positive and negative emotions and implemented more coping strategies to maintain resilience. Altruism, including a sense of sacred mission and self-worth, is essential for the maintenance of resilience among ENs. Consistent with a previous study (23), our data showed that psychological resilience is not a stable psychological characteristic but a process that is constantly changing according to internal and external factors. Psychological resilience seems to be of particular importance and is being advocated as a panacea for nurses to successfully cope with adversity. However, it seems that more research is needed to explain psychological resilience not only as a process of coping with adversity but also as a competency that can be achieved through support mechanisms (e.g., organizational support and social support) (40).

The first theme that emerged from the study was risk factors. Effective organizational support is critical to reducing risk factors and protecting the physical and psychological well-being of ENs. Organizational support is a type of support that organizations give to their employees in terms of resources, guidance, encouragement, and communication so that they can more efficiently complete their work tasks (41). Many studies reported a correlation between organizational support, nurse performance (e.g., job performance, job satisfaction, creative thinking) and patients' feelings (e.g., patient satisfaction) (42). In addition, there is evidence that a higher level of organizational support may reduce the effect of workplace stressors and act as a promotion factor that increase nurses' resilience (43). In the early stages of the COVID-19 pandemic in Shanghai, ENs worked in unprecedentedly stressful and harsh environment. During the interviews, we also found that some ENs experienced physical discomforts, including diarrhea and sleep disturbances. The government of Shanghai has put enormous efforts to contain the epidemic, but the healthcare staff and economic development have paid a huge price.

First, on one side, based on lessons acquired during COVID-19, Ebola, and SARS, organizational managers should provide training opportunities for all healthcare staff to improve their responses to public health emergencies (44, 45) and ensure adequate front-line reserves (e.g., nursing pool) and supplies before an outbreak. The purpose of this study was not only to help ENs improve their emergency care skills but also to reserve talent in emergency response. On the other side, Lam et al. (44) reported that during an outbreak, ENs are often given new and unusual roles that may go beyond nurses' previous practice and raise ambiguity of roles. Our study also found that TNs were often unfamiliar or were not well-adapted to the work environment and work rhythm in ED when participating in rescue. In China, the professional competence of nurses was categorized as N0 (trainee), N1 (advanced beginner), N2 (competent), N3 (proficient), and N4 (expert) according to Benner's theory (46); thus, it is recommended that organizational managers should strengthen the supervision of support nurses and prioritize N2 or higher levels of nurses with emergency experience to support the work of ED. Meanwhile, organizational managers should further rationalize resources through walk-around management and ensure that each healthcare facility can communicate accurately and quickly. Effective organizational support, including timely training, defining the role of EN, reasonable resource allocation, and quick communication, might significantly avoid risk factors such as the stress of screening infected patients and unfamiliarity of TNs with the process.

Secondly, based on our interviews, prolonged use of personal protective equipment (PPE) can restrict movement, affect the diet, and cause fatigue and facial pressure marks. PPE limits nurses' face-to-face and eye contact with patients and colleagues. These issues seem to be addressed by restricting shift duration, offering adequate rest time, and detailed guidelines for using PPE (47). A reasonable workload is beneficial (even the most resilient are exhausted by long periods of understaffed and overwork) (48). The organizational support mentioned above would avoid the sudden multiplication of workload of ENs and contribute to maintaining resilience.

The second theme that emerged from the study was promoting factors. Management assurance and humanistic care are essential to promote resilience. ENs who experience public health emergencies are at great risk of experiencing traumatic events, such as exposure to workplace violence, the experience of patient death, infection of a colleague with COVID-19, and vicarious traumatization (VT) (49). ENs develop resilience when experiencing various forms of trauma, although they experience negative effects (50). However, too much of trauma can prevent growth in individuals (33). Effective organizational support should give ENs both external and emotional resources to cope with trauma. Therefore, creating a trustworthy work atmosphere of self-expression, understanding, and acceptance is especially important. In addition, the physical and psychological rewards ENs received from the organization were also important promoting factors in increasing their resilience. Organizational managers and health system stakeholders should reward front-line nurses at every opportunity.

It is believed that social support from external of the workplace (e.g., friends, family) and internal (e.g., peers, leaders) is essential for ENs to maintain resilience during adversity (51). It is consistent with the results of our research. Meanwhile numerous studies (43, 52, 53) have indicated that social support affects work satisfaction, career dedication, health, and well-being of nurses. Thus, it is essential to implement measures to enhance social support in the workplace. First, nurse leaders must find time to listen to and address nurses' comments and concerns. Active listening is a practical strategy that helps ENs gain psychological support and enhance their resilience (24). Second, it is necessary to encourage individuals with high resilience to help individuals with low resilience to increase their psychological resilience through role modeling and communication. Third, Employee Assistance Program (EAP) is considered effective (54, 55). However, using EAP in China's healthcare industry is not common. Only a handful of hospitals have implemented a simplified service for a small number of nurses, and no institutionalized system has been formed yet (55). Finally, the family status of ENs should be taken seriously, and appropriate help should be given.

This study found that ENs' recognition of adversity and resilience and self-control were critical to coping with adversity. Evidence suggests that personal characteristics such as being well-adapted, self-control, positive attitude, hope and self-efficacy are important factors for high resilience in nurses (24). Furthermore, we believe that resilience is not just the individual's responsibility. Rather, resilience is a shared responsibility between individuals and teams. Team resilience is a dynamic psychological process of team members based on shared coping beliefs, expressed as a collective psychological state of shared cognition, shared motivation, or shared emotions of team members. When team adversity is perceived, team members call on their positive psychological resources and extend individual resilience to the team level through interpersonal interactions. Team resilience also acts as a protective factor for individual psychological resilience (56). Especially in ED, which primarily relies on multidisciplinary teams to provide patient care, team resilience is crucial. First of all, simulation training can boost teamwork's confidence and improve team resilience, such as simulation education and team management of clinical aggression training (57). Secondly, when experienced team leaders have the ability to clearly communicate and organize team operations, team members feel absolutely supported, which in turn increases their confidence. Team resilience course training for team leaders could be taken after adversity (58). Again, online platforms (e.g., WeChat, QQ, Facebook) can be used to achieve an information-sharing platform and create a good team support atmosphere. As mentioned before, participants played games or comforted each other online, which effectively relieved work stress.

**Figure 2:**
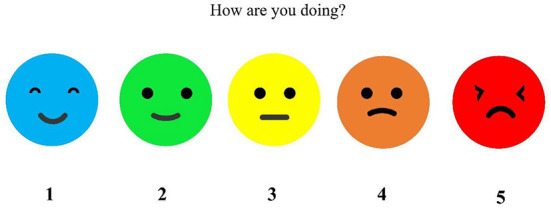
Employee mood meter.

Consideration needs to be given to ENs with low psychological resilience. It is also necessary to protect the team resilience from the bad emotions and negative impact that these nurses may produce. Taylor (59) used three levels addressing nurse resilience: primary, secondary, and tertiary. Primary interventions focus on building resilience through coping skills and communication skills; secondary interventions assess ENs experiencing burnout and give corresponding support to eliminate burnout; tertiary interventions should target ENs that exceed resilience thresholds and give treatment to return to work safely. To make judgments, nurse leaders can measure the current emotional state of ENs and encourage them to express their feelings openly. This can be done, for example, by using a mood meter during the daily morning meeting or Wechat voting (Figure 2) (60). It is recommended that hospital management arranges timely counseling and training focused on psychological stress knowledge and self-adjustment skills by a psychiatrist. Psychological preparation can lessen the initial anxiety and fear of entry (38). In addition, resilience training interventions using mindfulness and Cognitive Behavioral Therapy (CBT) techniques can strengthen resilience among ENs. Also, Stress Management and Resilience Training (SMART) (61) can be used to manage stress and improve the resilience of nurses. It has been demonstrated that a multimodal resilience training program is highly beneficial in helping nurses build effective coping skills and increase resilience (62).

The final theme that emerged from the study was motivated altruism. Many studies (37, 63) showed that the epidemic of COVID-19 can be stressful for nurses. Our findings also indicated that COVID-19 causes heavy stress and psychological distress, but ENs developed psychological resilience. The desire to help others during an epidemic also appears to be associated with altruism, which is understood as disinterested love. The phenomena of public showing thanks to “masked heroes” in Shanghai, China, and throughout the globe have inspired ENs and harnessed their goal orientation to respond to the epidemic. In this process, ENs achieved self-worth and awakened positive qualities, such as a sense of sacred mission. In addition, nurse empowerment should be supported. It is necessary to encourage more nurses with acute care experience to engage in public health emergency decision-making, free of bureaucratic restrictions. For example, ENs with relevant experience should select the type of PPE for staff. When ENs feel supported and valued for their expertise, their self-worth and resilience increase.

## Study limitations

The current study has several limitations. Seventeen participants were ENs; due to the inherent nature of qualitative research, the results of this study cannot be generalized to all healthcare professionals. The experiences of healthcare providers and managers other than ENs should be explored further. Furthermore, this was short-term research. The long-term experience of the research participants will serve as a great resource for future research, which means we will continue this research in depth in the future.

## Conclusion

COVID-19 has brought profound devastation to the world, and it is not known when it will end (1). Whether in the current situation or in future epidemics, ENs always have the important task of first-line care and occupy a central position in public health emergency. This study provides a comprehensive and in-depth understanding of the psychological resilience of ENs during COVID-19 through a phenomenological approach. We found that positive and negative emotions in ENs coexisted during the outbreak. Management assurance and humanistic care, social support, and positive coping are promoting factors for ENs to maintain psychological resilience. The risk factors, such as sudden multiplication of workload, the stress of identifying infected patients, and the unfamiliarity of support nurses, should lead to a deep reflection on the management organizers. This study provides basic data for further psychological interventions. The novelty of this study is that the findings can be used to improve ENs' preparedness in the face of a sudden public health emergency, such as COVID-19, and to maintain nurses' resilience in adversity. This study emphasizes the positive aspects of ENs facing adversity, such as awakening of a sense of sacred mission and realization of self-worth (e.g., receiving respect from society). These qualities should be preserved and reinforced, not only for the “heroes” of this emergency, but also for the global health care of tomorrow.

## Data availability statement

The original contributions presented in the study are included in the article/supplementary material, further inquiries can be directed to the corresponding author/s.

## Ethics statement

The studies involving human participants were reviewed and approved by the Institutional Review Committee of Shanghai Tenth People's Hospital granted ethical. The patients/participants provided their written informed consent to participate in this study. Written informed consent was obtained from the individual(s) for the publication of any potentially identifiable images or data included in this article.

## Author contributions

JJ and XD: study design. YL, HP, PH, PZ, and HS: data collection, data analysis, and manuscript preparation. All authors contributed to the article and approved the submitted version.

## Funding

This work was supported by Shanghai Shenkang Hospital Development Center Clinical Science and Technology Innovation Project (SHDC12021611) and Shanghai Medical Union Theory Research Key Project (2022YGL10).

## Conflict of interest

The authors declare that the research was conducted in the absence of any commercial or financial relationships that could be construed as a potential conflict of interest.

## Publisher's note

All claims expressed in this article are solely those of the authors and do not necessarily represent those of their affiliated organizations, or those of the publisher, the editors and the reviewers. Any product that may be evaluated in this article, or claim that may be made by its manufacturer, is not guaranteed or endorsed by the publisher.
